# Coffee Pulp and Silverskin Mitigate Fructose-Induced Intestinal Alterations in Rats

**DOI:** 10.3390/biom16071069

**Published:** 2026-07-22

**Authors:** Francisca Silva, Nelson Andrade, Ilda Rodrigues, Cláudia Marques, Juliana A. Barreto-Peixoto, Maria B. P. P. Oliveira, Rita C. Alves, Fátima Martel

**Affiliations:** 1 Unit of Biochemistry, Department of Biomedicine, Faculty of Medicine of Porto, University of Porto, 4200-319 Porto, Portugal; a2023534@nms.unl.pt (F.S.); nandrade@med.up.pt (N.A.); irodrigues@med.up.pt (I.R.); 2 Nutrition & Metabolism, Comprehensive Health Research Centre (CHRC), NOVA Medical School, Faculdade de Ciências Médicas, Universidade NOVA de Lisboa, 1169-056 Lisboa, Portugal; claudia.sofia.marques@nms.unl.pt; 3 Laboratório Associado para a Química Verde—Tecnologias e Processos Limpos (REQUIMTE/LAQV), Department of Chemical Sciences, Faculty of Pharmacy, University of Porto, 4050-313 Porto, Portugal; jabpeixoto95@gmail.com (J.A.B.-P.); beatoliv@ff.up.pt (M.B.P.P.O.); rita.c.alves@gmail.com (R.C.A.); 4 Instituto de Investigação e Inovação em Saúde (i3S), University of Porto, 4050-313 Porto, Portugal

**Keywords:** fructose, coffee pulp, coffee silverskin, intestine, sugar absorption, sugar sensing, microbiota

## Abstract

Excessive fructose consumption is associated with metabolic syndrome (MS). This study evaluated the effect of two coffee by-products, coffee pulp (CP) and coffee silverskin (SK), on fructose-induced intestinal changes. Sprague–Dawley rats were assigned to six groups (*n* = 6/group) for 10 weeks: Control, Fructose (FRU; 20% fructose in drinking water), CP, CP + FRU, SK, and SK + FRU. CP and SK were administered by oral gavage (250 mg/kg/day) using corn oil as vehicle. Intestinal morphology, gene expression (RT-qPCR), and gut microbiota composition (16S rRNA sequencing) were assessed. Fructose significantly increased jejunal expression of the glucose transporters *SGLT1* and *GLUT2*. CP and SK reversed *SGLT1* and *GLUT2* overexpression and reduced *GLUT5* expression relative to the FRU group. Fructose also markedly increased expression of sweet taste receptors *TAS1R2* and *TAS1R3* and the transcription factors *SREBP-1c* and *ChREBP*. Both CP and SK normalized *TAS1R2* and *TAS1R3* expression, whereas SK additionally prevented *SREBP-1c* and *ChREBP* overexpression. Both by-products restored fructose-induced reductions in microbial richness and alpha diversity. CP also modified beta diversity and increased the abundance of the genus *Blautia* compared with FRU. In conclusion, CP and SK reversed several fructose-induced intestinal alterations, namely in the jejunal expression of sugar-sensing and absorption-related genes. Additionally, CP showed microbiota-modulating effects, whereas SK modulated the jejunal expression of key transcription factors (*SREBP-1c* and *ChREBP*) involved in carbohydrate and lipid metabolism. Overall, these findings suggest that CP and SK may represent promising candidates for mitigating fructose-induced intestinal alterations.

## 1. Introduction

Metabolic syndrome (MS) is a cluster of metabolic risk factors (hyperglycemia, insulin resistance, hypertension, hypertriglyceridemia, low high-density lipoprotein cholesterol and abdominal obesity) that tend to occur together, significantly increasing the risk of type 2 diabetes, cardiovascular diseases, metabolic dysfunction-associated steatotic liver disease and some types of cancer [1,2]. MS is a major contributor to premature morbidity and mortality, being a growing global health concern [3]. MS is primarily driven by genetic predisposition and lifestyle factors (sedentary lifestyle and obesogenic dietary habits), and its current major treatment options center on combining intensive lifestyle modifications (such as a healthy diet, weight loss, and regular physical activity) with targeted pharmacotherapies to manage individual issues like high blood pressure, elevated cholesterol, and insulin resistance, and bariatric surgery for severe cases [4]. Modern diets are characterized by a high intake of fructose, in the form of sucrose and high-fructose corn syrup (a common sweetener used in the food industry), and high intake of this sugar is linked to MS development, both in rodent experimental models [5,6] and human populational studies [7,8].

Coffee is among the most widely consumed beverages globally, resulting in the large-scale generation of industry by-products, including coffee pulp (CP) and coffee silverskin (SK), which present both environmental challenges and opportunities for sustainable valorization [9,10,11]. CP (the outer part of the coffee cherry) is the first by-product of the coffee wet post-harvesting processing and represents about 40–50% of the fresh coffee berry weight [12]. On the other hand, SK (a thin tegument that covers the green coffee beans) is the major by-product of coffee roasting companies [13]. Although generally discarded, both CP and SK have been highlighted as a natural source of dietary fiber, protein and bioactive compounds that can be further used, directly or after extraction, in the development of several functional food products [9,10,11,14].

Coffee has garnered special attention in the context of MS because of its beneficial impact on metabolism [15]. Although research on the possible benefits of coffee on MS has focused on roasted coffee as a beverage, intermediary products such as CP and SK are also rich in the same bioactive compounds found in the coffee beverage, as mentioned above. Interestingly, our group recently verified that consumption of CP ameliorated several MS-associated consequences of high-fructose intake in rats. Namely, CP ameliorated the deleterious effects of fructose consumption on body weight, blood pressure, fasting plasma glucose, insulin, leptin, TAG and inflammatory marker levels and on insulin resistance [16,17]. Moreover, at the hepatic level, CP abolished the increase in fat and glycogen accumulation induced by fructose and had a beneficial effect on oxidative stress-related parameters [17]. Although less effective, consumption of SK was also found to have beneficial effects on several changes induced by fructose feeding in the same animal model [16].

The intestine plays a central role in MS—and particularly in fructose-induced MS—as the primary site where excessive fructose is absorbed and metabolized and where it interacts with the gut microbiota and intestinal epithelium, leading to microbiota shifts and impaired barrier function (“leaky gut”) that contribute to systemic inflammation, insulin resistance and metabolic dysfunction [18,19].

Given the important role of the intestine in fructose-induced MS, the aim of this work was to explore the influence of CP and SK at this level in fructose-fed animals, as a factor contributing to their beneficial effects upon fructose-induced MS. In detail, mechanisms by which CP and SK may modulate fructose-induced intestinal changes were explored by analyzing intestinal morphologic, metabolic and microbiota characteristics.

## 2. Materials and Methods

### 2.1. In Vivo Experiments

#### 2.1.1. Animals

Thirty-six male adult (7 weeks old) Sprague–Dawley rats (250–315 g body weight) were obtained from Charles River Laboratories (Saint Germain Nuelles, France). Upon arrival, rats were housed in pairs, in a temperature-controlled (20–22 °C) room on a 12:12 h dark–light cycle. Before the experimental procedure, animals were allowed an acclimation period of 7 days in the environment with free access to standard laboratory diet (diet #4RF21 certificate, Mucedola, Milan, Italy) and tap water. The chemical composition of the feed is shown in Supplementary Table S1. Animals were handled and cared for according to the Council of Europe guidelines for the use of laboratory animals (86/609/EEC) and Law 129/92. The experimental protocol was approved by the Animal Welfare and Ethics Committee (ORBEA) of the Faculty of Medicine of the University of Porto, Portugal, and by the Directorate General of Food and Veterinary of the Portuguese Government (0421/000/000/2022).

#### 2.1.2. Experimental Design

MetS was induced in normoglycemic rats by replacing tap water with tap water containing 20% (*w*/*v*) D-fructose (VWR International, Radnor, PA, USA) for 10 weeks. Fructose solution was freshly prepared every alternate day and administered *ad libitum*. Dried CP from Costa Rica (Alajuela region) was acquired from the coffee roaster company TADAH kafferosteri AB (Lenhovda, Sweden) and dried SK was kindly provided by Torrié (JMV Group, Rio Tinto, Portugal). Samples (~1 kg each) were ground and stored as described [16,17]. The chemical analysis of CP and SK is shown in Supplementary Table S1. CP and SK were administered daily by gavage (250 mg/kg b.w.; mixed in 0.25 mL corn oil/kg rat weight; this solution was freshly prepared every day). Their controls were administered 0.25 mL corn oil /kg b.w. daily (by gavage). The dose of CP and SK administered translates to a Human Equivalent Dose of ≈40.5 mg/kg/day, which closely mirrors the standard commercial daily serving sizes (2.5–3.0 g/day) of dehydrated whole coffee fruit and SK dietary fiber powders sold in the nutraceutical and functional food markets. On the other hand, this daily dose of CP and SK contributes a maximum of 0.244 and 0.212 kcal/day, respectively (as gross energy of CP and SK is ≈2.4 kcal/g and 2.12 kcal/g, respectively), which represents a negligible fraction (0.3–0.5%) of the total daily caloric intake. So, no major dietary energy shifts occurred in these animals because of CP or SK ingestion.

The thirty-six male rats were randomly divided into six groups, with each group comprising six animals, as follows:

(I) Control (CONT): this group received *ad libitum* standard diet and tap water + corn oil (0.25 mL/kg b.w./day);
(II) Coffee pulp (CP): this group received *ad libitum* standard diet and tap water + CP (250 mg/kg b.w./day in 0.25 mL corn oil/kg b.w./day);
(III) Silverskin (SK): this group received *ad libitum* standard diet and tap water + SK (250 mg/kg b.w./day in 0.25 mL corn oil/kg b.w./day);
(IV) Fructose (FRU): this group received *ad libitum* standard diet and tap water supplemented with 20% (*w*/*v*) fructose + corn oil (0.25 mL/kg b.w./day);
(V) Fructose + Coffee pulp (FRU + CP): this group received *ad libitum* standard diet and tap water supplemented with 20% (*w*/*v*) fructose + CP (250 mg/kg b.w./day in 0.25 mL corn oil/kg b.w./day);
(VI) Fructose + Silverskin (FRU + SK): this group received ad libitum standard diet and tap water supplemented with 20% (*w*/*v*) fructose + SK (250 mg/kg b.w./day in 0.25 mL corn oil/kg b.w./day)

Sample size was determined based on our previous experience with this animal model [20,21] and consideration of ethical principles to minimize animal use while maintaining sufficient statistical power for the primary study outcomes. No formal *a priori* power analysis was performed.

At the end of the 10th week of treatment, and after a 6 h fasting period, the animals were deeply euthanized with sodium phenobarbital (100 mg/kg b.w., i.p., Euthanimal, Nephar) and blood was collected from the left ventricle into heparinized needles. Then, rats were transcardially perfused with ice-cold isotonic sodium chloride solution. After perfusion, a segment of the duodenum (immediately distal to the pyloric sphincter) and of the jejunum (10 cm distal to the pyloric sphincter) were excised for histological analysis (see below). Another segment of the jejunum was also removed, and the mucosa was scraped with a sterile scalpel, immediately immersed in liquid nitrogen and stored at −80 °C for further use in RT-qPCR. Moreover, stool samples were collected from the distal colon and stored at −80 °C.

#### 2.1.3. Histological Analysis

Duodenum and jejunum samples were first fixed in neutral-buffered formaldehyde aqueous solution (10%) for 48 h at 4 °C and then dehydrated and embedded in paraffin. Paraffin sections (3 μm) were cut with a microtome (Leica^®^ RM2125RT; Leica Microsystems, Vila Nova de Famalicão, Portugal) and then stained with hematoxylin and eosin (H&E) (Sigma-Aldrich, St. Louis, MO, USA). Four random images of each sample were captured (40× amplification; Nikon Eclipse E50i light microscope, Nikon Corporation Instruments Company, Tokyo, Japan) and analyzed using ImageJ software (version 2.14.0, National Institutes of Health, Bethesda, MD, USA). In total, 10 villi and 10 crypts from each animal were analyzed, and the following parameters were measured—villus height, villus width, crypt depth—as described [22,23]. Villi height/crypt depth ratio was also determined.

#### 2.1.4. RT-qPCR

Total RNA was isolated from the jejunum mucosa using NZYol reagent (NZYTech, Lisbon, Portugal) according to the manufacturer’s instructions, and the concentrations were determined by a NanoDrop One (Thermo Fisher Scientific, Waltham, MA, USA). Conversion of 1 μg total RNA into cDNA was done using a qScript cDNA SuperMix (Quanta Biosciences, Beverly, MA, USA), following the manufacturer’s instructions.

Quantitative real-time polymerase chain reaction (RT-qPCR) was then run on a Lightcycler 96 System (Roche, Mannheim, Germany) utilizing the KAPA SYBR FAST qPCR master mix kit (Kapa Biosystems, Wilmington, MA, USA), RNase-free water, and forward and reverse primers (100 mM stock, final concentration 10 mM) at a 5:3.6:0.2 ratio following the manufacturer’s instructions. The sequences and annealing temperatures of primers (Invitrogen, Thermo Fisher Scientific, Reinach, Switzerland) are listed in Supplementary Table S2.

The cycling conditions for *SGLT1*, *GLUT2* and *GLUT5* were as follows: denaturation (95 °C for 10 min), amplification and quantification (95 °C for 10 s, annealing temperature for 10 s and 72 °C for 10 s, with a single fluorescence measurement at the end of the 72 °C for a 10 s segment) repeated for 50 cycles, and a final melting step with a temperature ramp from 60 °C to 97 °C [20]. The cycling conditions for *TAS1R2* and *TAS1R3* were as follows: denaturation (95 °C for 10 min), amplification, and quantification (60 °C for 5 s, annealing temperature for 30 s and 72 °C for 30 s, with a single fluorescence measurement at the end of the 72 °C for a 10 s segment) repeated for 50 cycles and a final melting step with a temperature ramp from 60 °C to 97 °C [21]. The cycling conditions for *SREBP-1c* and *ChREBP* were as follows: denaturation (95 °C for 10 min), amplification, and quantification (95 °C for 10 s, annealing temperature for 30 s and 72 °C for 30 s, with a single fluorescence measurement at the end of the 72 °C for a 10 s segment) repeated for 50 cycles and a final melting step with a temperature ramp from 60 °C to 97 °C [17]. Glyceraldehyde-3-phosphate dehydrogenase (*GAPDH*) was used as an endogenous (housekeeping gene) control. The 2^−ΔΔCT^ method [24] was used to determine the relative expression of the target genes normalized to glyceraldehyde 3-phosphate dehydrogenase (GAPDH).

#### 2.1.5. Gut Microbiota Analysis

Genomic DNA was extracted from fecal samples of all rat groups using the NZY Tissue Genomic DNA Isolation Kit (Nzytech, Lisbon, Portugal) [25] and quantified with a NanoDrop spectrophotometer (Thermo Scientific, Wilmington, DE, USA). The V3–V4 region of the bacterial 16S rRNA gene was amplified using universal primers 341F (5′-CCTAYGGGRBGCASCAG-3′) and 806R (5′-GGACTACNNGGGTATCTAAT-3′) with Phusion^®^ High-Fidelity PCR Master Mix (New England Biolabs, Ipswich, MA, USA). PCR conditions consisted of an initial denaturation at 98 °C for 1 min, followed by 30 cycles of denaturation at 98 °C for 10 s, annealing at 50 °C for 30 s, extension at 72 °C for 30 s, and a final extension at 72 °C for 5 min. PCR products were verified by agarose gel electrophoresis, and sequencing libraries were prepared according to the manufacturer’s instructions. Libraries were sequenced on the Illumina NovaSeq 6000 platform (Illumina, San Diego, CA, USA) to generate 250 bp paired-end reads.

Paired-end reads were demultiplexed using unique barcodes and trimmed to remove barcodes and primer sequences. Adapters were removed with cutadapt (v3.3), and reads were merged using FLASH (v1.2.11) [26]. Quality filtering was performed with fastp (v0.23.1) to obtain high-quality clean tags [27]. Chimeric sequences were identified against the SILVA (16S/18S) and UNITE (ITS) reference databases and removed using vsearch (v2.16.0) [28].

Denoising was performed using DADA2 to generate Amplicon Sequence Variants (ASVs), providing higher sensitivity, specificity, and reproducibility than traditional OTU-based methods [29,30,31]. Taxonomic classification of ASVs was conducted in QIIME2 v2024.10 using the *classify-sklearn* Naive Bayes classifier with the SILVA 138.1 database as reference [32,33].

#### 2.1.6. Statistical Analysis

Morphometric and RT-qPCR results are presented as arithmetic means ± SEM. Statistical analysis of the differences between the groups of animals was evaluated by one-way analysis of variance (ANOVA), followed by Tukey’s multiple comparisons *post hoc* test. The analysis was done using GraphPad prism version 10.0 (GraphPad Software Inc., San Diego, CA, USA). Differences were considered significant when *p* ≤ 0.05.

Microbiota data were analyzed using MicrobiomeAnalyst 3.0, an online platform for microbiome statistical and visual analyses [33]. All Amplicon Sequence Variants (ASVs) were included. No data normalization available (data transformation, rarefaction, or data scaling) was applied. Rarefaction curves confirmed adequate sequencing depth, suggesting that sequencing effort was adequate for downstream diversity analyses. Alpha diversity was assessed using Chao1 and Shannon indices [34]. Comparisons between groups were performed using Student’s *t*-test or one-way ANOVA, followed by *post hoc* multiple comparison tests as appropriate. Beta diversity was evaluated by PCoA based on Bray–Curtis dissimilarity and PERMANOVA [35]. Comparisons of taxa abundance between groups were performed by one-way ANOVA. Differences were considered statistically significant when *p* < 0.05 and *q* < 0.05 (after adjustment for multiple comparisons). Multivariate adjustment was based on the Benjamini–Hochberg procedure.

## 3. Results

### 3.1. The Effect of Fructose and/or Coffee By-Products on Duodenum and Jejunum Mucosa Morphology

**Figure 1:**
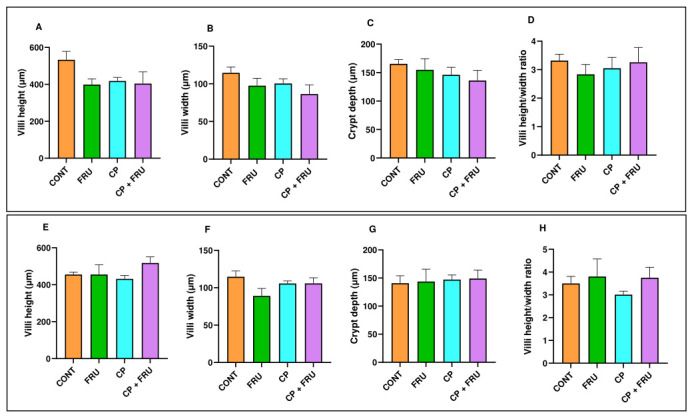
Duodenum (**A**–**D**) and jejunum (**E**–**H**) villi height, villi width, crypt depth and villi height/crypt depth ratio, after 10 weeks of dietary intervention, in Control (CONT), Fructose (FRU), Coffee Pulp (CP) and Coffee Pulp + Fructose (CP + FRU) groups. Hematoxylin and eosin (H&E) stained sections were used to measure villi height (10 villi/rat) (**A**,**E**), villi width (10 villi/rat) (**B**,**F**), crypt depth (10 open crypts/rat) and villi height/crypt depth ratio (**C**,**G**). Results are shown as arithmetic means ± SEM (*n* = 6 animals per group).

**Figure 2:**
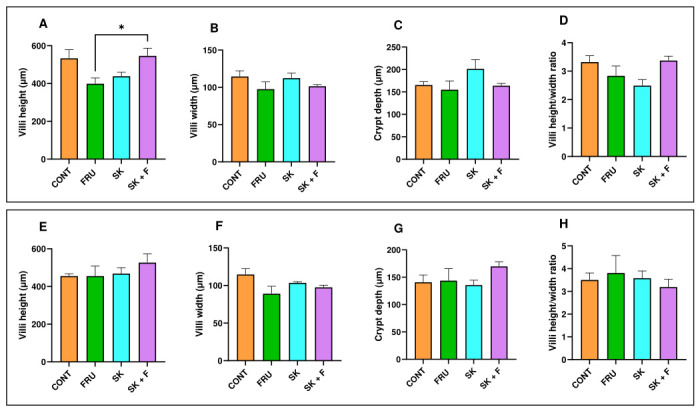
Duodenum (**A**–**D**) and jejunum (**E**–**H**) villi height, villi width, crypt depth and villi height/crypt depth ratio, after 10 weeks of dietary intervention, in Control (CONT), Fructose (FRU), Coffee Silverskin (SK) and Coffee Silverskin + Fructose (SK + FRU) groups. Hematoxylin and eosin (H&E) stained sections were used to measure villi height (10 villi/rat) (**A**,**E**), villi width (10 villi/rat) (**B**,**F**), crypt depth (10 open crypts/rat) and villi height/crypt depth ratio (**C**,**G**). Results are shown as arithmetic means ± SEM (*n* = 6 animals per group). * Significantly different from CONT (*p* < 0.05).

As shown in Figure 1 and Figure 2, neither fructose nor CP or SK, either alone and in combination, had any significant effect on duodenal and jejunal villi width and height, crypt depth and villi heigh/width ratios. The exceptions were a higher villi height in SK + FRU than in FRU animals and a non-significant tendency for a lower duodenal villi height (*p* = 0.067) and jejunal villi weight (*p* = 0.052) in FRU animals than in CONT animals.

### 3.2. The Effect of Fructose and/or Coffee By-Products on Sugar Sensing, Absorption and Metabolism

Next, we investigated the putative influence of the dietary interventions on the expression levels of genes coding for intestinal sugar sensing, absorption and metabolic adaptation.

**Figure 3:**
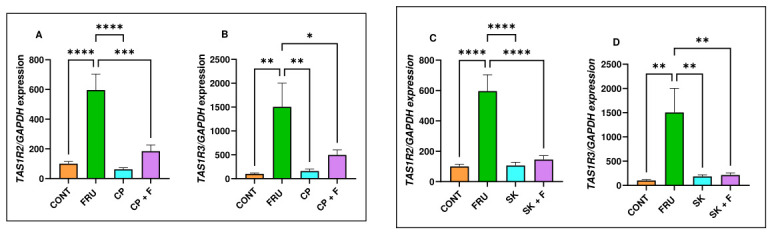
Quantification of mRNA levels of (**A**,**C**) Taste Receptor Type 1 Member 2 (*TAS1R2*) and (**B**,**D**) Taste Receptor Type 1 Member 3 (*TAS1R3*), by RT-qPCR, in jejunum mucosa of Control (CONT), Fructose (FRU), Coffee Pulp (CP), Coffee Silverskin (SK), Coffee Pulp + Fructose (CP + FRU) and Coffee Silverskin + Fructose (SK + FRU) groups (*n* = 6 animals per group). Results are shown as the expression of *TAS1R2* or *TAS1R3* relative to GAPDH (arithmetic means ± SEM). * The difference between groups was considered statistically significant (* *p* < 0.05, ** *p* < 0.01, *** *p* < 0.001, **** *p* < 0.0001).

Fructose consumption was found to cause a very marked increase in the mRNA levels of the sweet taste receptors *TAS1R2* and *TAS1R3* (to ±6× and 15× the CONT levels, respectively). Interestingly, these levels returned to CONT levels when either CP or SK was simultaneously present (Figure 3).

**Figure 4:**
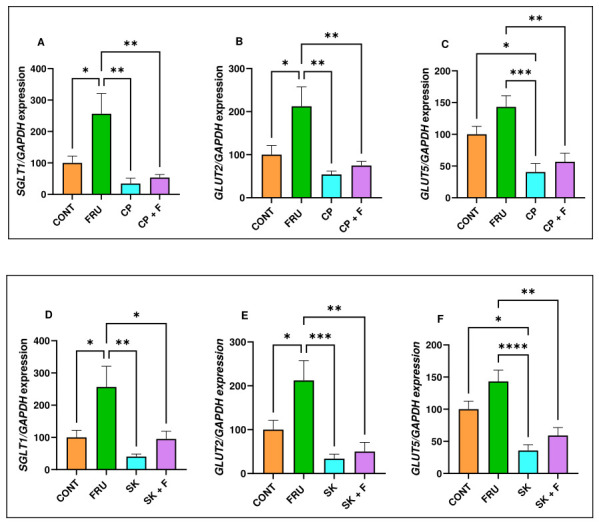
Quantification of mRNA levels of (**A**,**D**) sodium-glucose-linked transporter 1 (*SGLT1*), (**B**,**E**) facilitative glucose transporter 2 (*GLUT2*) and (**C**,**F**) facilitative glucose transporter 5 (*GLUT5*), by RT-qPCR, in jejunum mucosa of Control (CONT), Fructose (FRU), Coffee Pulp (CP), Coffee Silverskin (SK), Coffee Pulp + Fructose (CP + FRU) and Coffee Silverskin + Fructose (SK + FRU) groups (*n* = 6 animals per group). Results are shown as the expression of SGLT1, GLUT2 or GLUT5 relative to GAPDH (arithmetic means ± SEM). * The difference between groups was considered statistically significant (* *p* < 0.05, ** *p* < 0.01, *** *p* < 0.001, **** *p* < 0.0001).

The expression levels of the most important intestinal sugar transporters (*SGLT1*, *GLUT2* and *GLUT5*) are shown in Figure 4. In relation to *SGLT1*, high-fructose consumption (FRU) resulted in a marked increase in its mRNA levels (to ±2.5× the CONT levels), and co-ingestion of either CP or SK was able to completely abolish this increase (Figure 4A). The same trend was observed with *GLUT2*, as its gene expression level was greatly increased in FRU animals (to ±2× the CONT levels), and this increase was completely annulled in both CP + FRU and SK + FRU animals (Figure 4B). In relation to *GLUT5*, although FRU did not significantly affect *GLUT5* mRNA expression, a trend toward increased expression was observed. Importantly, *GLUT5* mRNA levels in CP + FRU and SK + FRU groups were significantly lower than those in the FRU group (Figure 4C). Together, we conclude that CP and SK were able to prevent fructose-induced increase in *SGLT1* and *GLUT2* and reduced *GLUT5* mRNA levels when co-administered with fructose.

**Figure 5:**
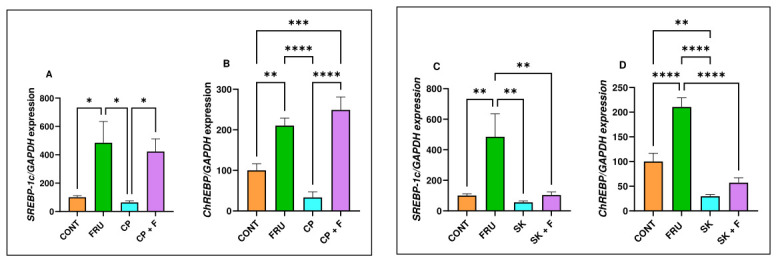
Quantification of mRNA levels of (**A**,**C**) Sterol Regulatory Element-Binding Protein 1c (*SREBP-1c*) and (**B**,**D**) Carbohydrate Response Element-Binding Protein (*ChREBP*) by RT-qPCR in jejunum mucosa of Control (CONT), Fructose (FRU), Coffee Pulp (CP), Coffee Silverskin (SK), Coffee Pulp + Fructose (CP + FRU) and Coffee Silverskin + Fructose (SK + FRU) groups (*n* = 6 animals per group). Results are shown as the expression of *SREBP-1c* or *ChREBP* relative to GAPDH (arithmetic means ± SEM). * The difference between groups was considered statistically significant (* *p* < 0.05, ** *p* < 0.01, *** *p* < 0.001, **** *p* < 0.0001).

In relation to the gene expression levels of the transcription factors *SREBP-1c* and *ChREBP*, we verified that treatment of the animals with fructose (FRU group) induced a marked increase in *SREBP-1c* (to ±5× the CONT levels) and *ChREBP* (to ±2× the CONT levels) mRNA levels (Figure 5). It was noticeable that the two coffee by-products had distinct effects upon *SREBP-1c* and *ChREBP* mRNA levels. Indeed, SK, but not CP, was able to mitigate the changes induced by FRU. Moreover, SK alone reduced the expression levels of *ChREBP* (Figure 5).

### 3.3. The Effect of Fructose and/or Coffee By-Products on Gut Microbiota

#### 3.3.1. Microbiota Abundance/Composition

**Figure 6:**
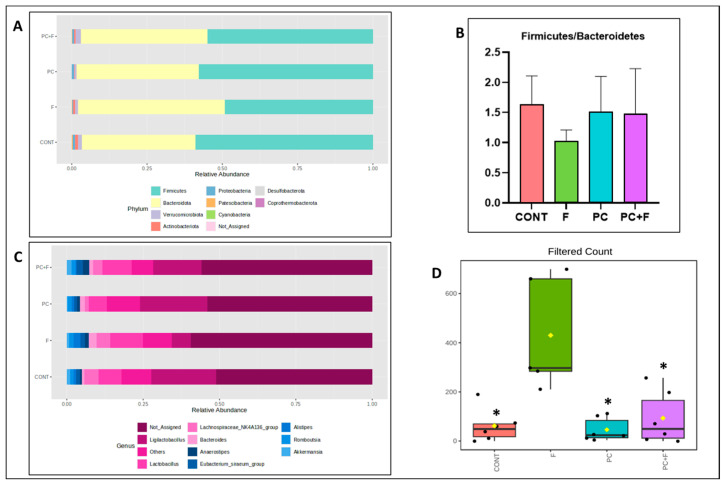
Analysis of intestinal microbiota in fructose- and/or CP-treated animals. (**A**) Intestinal microbiota composition at the phylum level, showing the most abundant phyla; (**B**) Firmicutes/Bacteroidetes ratio; (**C**) intestinal microbiota composition at the genus level, showing the most abundant genera; (**D**) box-and-whisker plot of the absolute abundance of *Blautia*, showing differences between groups (*p* < 0.05, *q* < 0.05). The line indicates the median, the box the interquartile range, and the whiskers the minimum and maximum values. Each point represents one animal. * *p* < 0.05 okvs. F (ANOVA with Tukey’s post hoc test). CONT: Control, F: Fructose, PC: Coffee pulp, PC + F: Coffee pulp + Fructose. Not_Assigned: the phyla or genera could not be determined. Shown are arithmetic means ± SEM (*n* = 6 animals per group).

**Figure 7:**
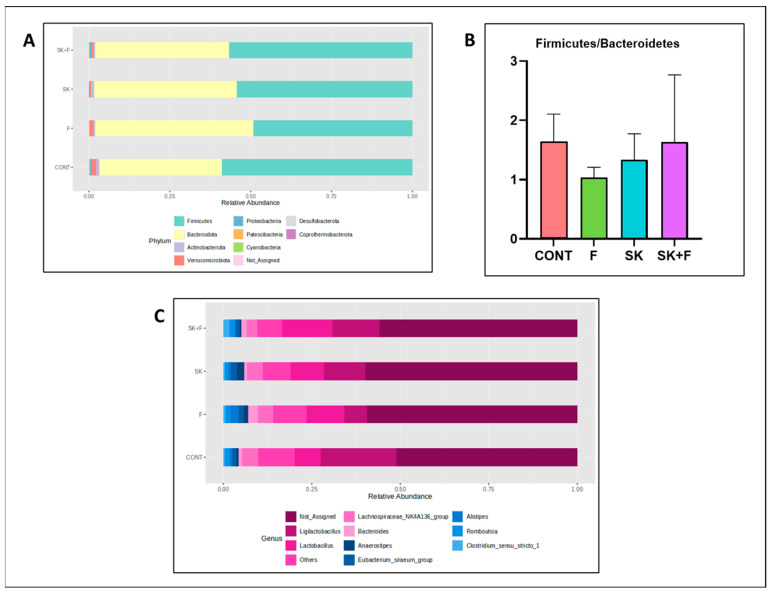
Analysis of intestinal microbiota in fructose- and/or CP-treated animals. (**A**) Intestinal microbiota composition at the phylum level, showing the most abundant phyla; (**B**) Firmicutes/Bacteroidetes ratio; (**C**) intestinal microbiota composition at the genus level, showing the most abundant genera. CONT: Control, F: Fructose, SK: Coffee silverskin, SK + F: Coffee silverskin + Fructose. Not_Assigned: the phyla or genera could not be determined. Shown are arithmetic means ± SEM (*n* = 6 animals per group).

Analysis of the microbiota in the six groups of animals shows that Firmicutes and Bacteroidetes are the most abundant phyla (from the 10 phyla identified) in all groups (Figure 6A and Figure 7A). When analyzing the Firmicutes/Bacteroides ratio, no statistically significant differences were found, although there was a tendency to a lower ratio in the FRU group (Figure 6B and Figure 7B). Figure 6C and Figure 7C show the most expressed genera in all the groups of animals. Although the abundance of *Ligilactobacillus* appears to be lower in the FRU group of animals (Figure 6B and Figure 7B), relative to all the others, after adjusting for multiple comparisons, the only significant difference among all the 77 identified genera was a reduction in the fructose-induced increase in the abundance levels of the *Blautia* genus that was observed in CP + FRU animals (Figure 6C).

#### 3.3.2. Microbiota Diversity

**Figure 8:**
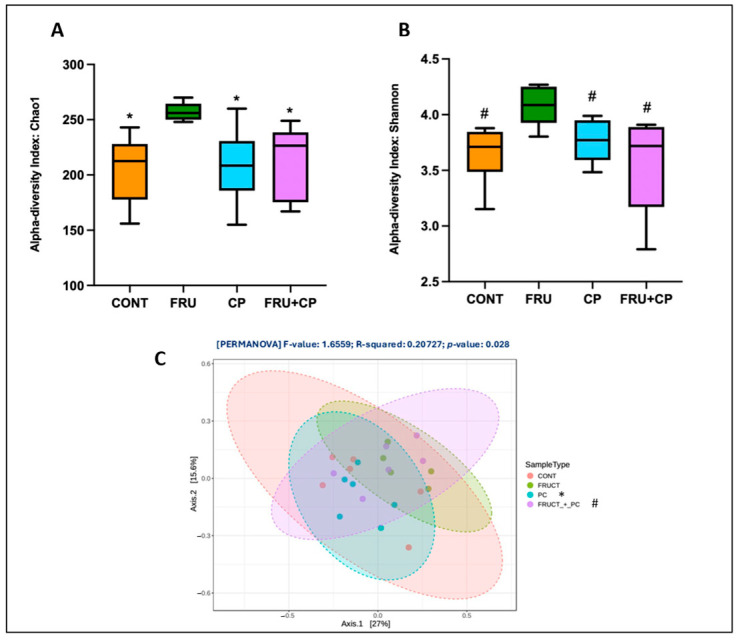
(**A**) Box-and-whisker plot of the Chao1 index (richness) across groups: control (CONT), fructose (F), coffee pulp (CP), and coffee pulp + fructose (CP + F) (*n* = 6 animals per group); (**B**) box-and-whisker plot of the Shannon index (diversity) for the same groups (*n* = 6 animals per group). In both panels, the central line represents the median, boxes indicate the interquartile range, whiskers show the minimum and maximum values, and each point represents an animal. * *p* < 0.05 and *q* < 0.05 vs. F for Chao1; # *p* < 0.05 and *q* = 0.058 vs. F for Shannon. (**C**) Principal coordinates analysis (PCoA) of bacterial community structure. Samples are plotted according to the first two principal coordinates (PCo1: 27%; PCo2: 15.6% of variance explained) and colored by group (CONT, F, CP, CP + F) (*n* = 6 animals per group). Each point represents an animal. * *p* < 0.001 and *q* < 0.05 for CP vs. F; # *p* < 0.05 and *q* = 0.081 for CP vs. CP + F.

**Figure 9:**
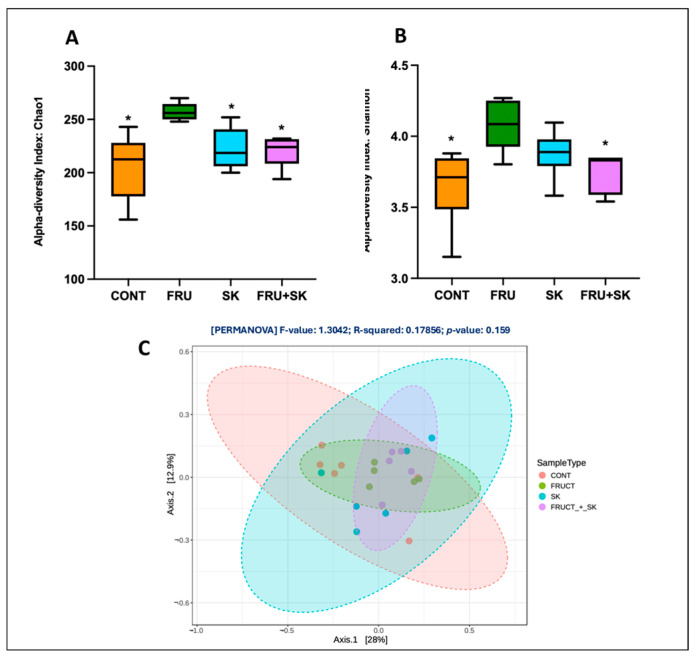
(**A**) Box-and-whisker plot of the Chao1 index (richness) across groups: control (CONT), fructose (F), coffee silverskin (SK), and coffee silverskin + fructose (SK + F) (*n* = 6 animals per group); (**B**) box-and-whisker plot of the Shannon index (diversity) for the same groups (*n* = 6 animals per group). In both panels, the central line represents the median, boxes indicate the interquartile range, whiskers show the minimum and maximum values, and each point represents an individual sample. * *p* < 0.01 and *q* < 0.05 vs. F for Chao1; * *p* < 0.05 and *q* < 0.05 vs. F for Shannon. (**C**) Principal coordinates analysis (PCoA) of bacterial community structure. Samples are plotted according to the first two principal coordinates (PCo1: 28%; PCo2: 12.9% of variance explained) and colored by group (CONT, F, SK, SK + F) (*n* = 6 animals per group). Each point represents an animal.

Analysis of the microbial community profile was done next. The results are shown in Figure 8A and Figure 9A (Chao1 index (richness)) and Figure 8B and Figure 9B (Shannon index (diversity)). By analyzing these figures, it is evident that the FRU group exhibits the highest richness (Chao1 index), whereas in both the CP + FRU (Figure 8A) and the SK + FRU (Figure 9A) groups, the effect of FRU alone is mitigated. Regarding diversity, as assessed by the Shannon index, the CP + FRU group showed a trend toward a lower diversity when compared to the FRU group, with values approaching those observed in the CONT group (Figure 8B), while the effect of FRU in this parameter was abolished in the SK + FRU group (Figure 9B).

Finally, beta diversity was assessed using principal coordinates analysis (PCoA) based on dissimilarity distances, enabling visualization of differences in microbial composition among CONT, FRU, CP, and CP + FRU groups (Figure 8C) and CONT, FRU, SK, and SK + FRU groups (Figure 9C).

Regarding CP, considerable overlap between groups was observed, although some separation trends were evident (Figure 8C). These findings suggest that dietary treatments influenced microbiota structure, although not sufficiently to produce clear clustering in the two-dimensional space. PERMANOVA revealed significant differences in microbial composition among groups, with dietary treatments explaining approximately 20% of the total variance. Pairwise comparisons identified the greatest difference between the FRU and CP groups (*p* < 0.001), while a trend toward difference was observed between CP and CP + F; CP remained similar to the CONT group. No significant differences in dispersion were detected among the experimental groups (*p* > 0.05), indicating that the observed PCoA separation reflects differences in community composition rather than within-group variability (Figure 8C).

In relation to SK, analysis of the beta diversity results shows that the distribution of samples showed substantial overlap among groups, consistent with the global PERMANOVA results, which indicated no statistically significant differences in microbial composition across dietary interventions (*p* = 0.159) (Figure 9C). Similarly, pairwise PERMANOVA comparisons revealed no statistically significant differences between any groups, after multiple testing correction using the Benjamini–Hochberg method (*p* > 0.05 for all comparisons) (Figure 9C).

## 4. Discussion

We recently verified that consumption of CP ameliorated several deleterious effects of high-fructose consumption in rats. Specifically, CP was able to ameliorate body weight gain, fasting hyperglycemia, hyperinsulinemia, hyperleptinemia, hypertriglyceridemia, hypertension, and increased inflammatory levels and to improve insulin resistance induced by fructose feeding [16,17]. Moreover, at the hepatic level, CP abolished the increase in fat and glycogen accumulation and oxidative stress levels induced by fructose [17]. Although less effective, consumption of SK was also found to have beneficial effects on several changes induced by fructose feeding. More specifically, it attenuated blood pressure elevation, improved glycemic control, reduced insulin resistance and ameliorated the increase in plasma inflammatory markers and in hepatic lipid peroxidation and glutathione levels in the same animal model [16].

Because the intestine plays a central role in fructose-induced MS [18,19], we decided to investigate if CP and SK are able to interfere with fructose-induced changes at the intestinal level, as a factor contributing to their beneficial effects upon fructose-induced MS.

The results of the morphometric study comparing duodenal and jejunal mucosa histology in the different groups of animals suggest that fructose and/or CP or SK feeding do not cause significant morphological alterations in jejunum and duodenal mucosa. Previous studies have reported variable effects of fructose on intestinal morphology. While two previous works concluded, similarly to us, that fructose consumption had no effect on rat jejunal villi and crypt structure [36,37], other studies found fructose to increase rat jejunal villus width [20], to enhance villus height, width, and crypt depth in rat distal ileum [38], and to increase villus length in mouse duodenum and proximal jejunum, which was associated with greater lipid absorption, weight gain, and fat accumulation [39]. A healthy mucosal architecture is vital not only for its absorptive capacity, but its crosstalk with the intestinal microbiota [40] is also an essential point to correlate with MS and obesity. So, more studies appear to be necessary in order to better elucidate the consequences of excessive fructose consumption on small intestinal mucosal morphology.

Absorption of dietary fructose is mediated by two transporters of the family of glucose transporters (GLUTs). GLUT5 (Slc2a5) is primarily responsible for fructose uptake into the enterocyte at the apical side of the membrane, whereas GLUT2 (Slc2a2) moves most of fructose from the cytosol into blood vessels at the basolateral side of the enterocyte [41,42]. GLUT2 has a high affinity for glucose and a moderate affinity for fructose, whereas GLUT5 is a fructose-specific transporter [43]. Moreover, absorption of fructose under a high-fructose diet also involves luminal GLUT2-mediated transport [44].

Several works have shown that chronic or acute fructose consumption increases SGLT1, GLUT2 and GLUT5 expression levels/activity in several animal models (e.g., rats and rabbits) and human proximal intestinal regions [43,45,46]. In agreement with this, fructose consumption in our animal model resulted in a significant increase in *SGLT1* and *GLUT2* mRNA levels, and a tendency for an increase in *GLUT5* mRNA levels. The lack of effect of fructose consumption on *GLUT5* mRNA levels may indicate that it modulates GLUT5 activity mainly by translational or posttranslational mechanisms, as was found in relation to dietary glucose-induced changes in small intestinal SGLT1 [46]. Importantly, both CP and SK were able to completely abolish fructose-induced increase in *SGLT1* and *GLUT2* mRNA levels, and additionally, they reduced *GLUT5* mRNA levels in fructose-fed animals. These results thus suggest that both CP and SK were able to mitigate the consequences of fructose feeding at the level of intestinal sugar transport.

The sweet taste receptor is a heterodimeric G-protein coupled receptor (GPCR) consisting of two subunits (TAS1R2/TAS1R3). Sweet taste receptors exist not only in gustatory tissues such as the tongue and palate, where they were originally described, but are also present in chemosensory cells in extra-oral tissues such as the gastrointestinal tract [47,48]. Natural sweeteners such as glucose or fructose and artificial sweeteners bind to TAS1R2/TAS1R3 that is coupled to a heterotrimeric G-protein (α-gustducin, Gβ3 and Gγ13) [44,48]. One important manifestation of sweet taste sensing by T1R2/T1R3 at the intestinal level is the regulation of sugar transporters. Indeed, activation of TAS1R2/TAS1R3 is known to increase SGLT1-, GLUT2- and GLUT5-mediated transport [46,47,48]. Distinct mechanisms appear to operate, including stimulation of TAS1R2/TAS1R3 in enterocytes that drives rapid GLUT2 translocation from intracellular vesicles to the apical membrane (via phospholipase C-β2 activation and increased Ca^2+^ levels) and stimulation of TAS1R2/TAS1R3 in enteroendocrine L-cells and K-cells that triggers incretin hormone release and subsequent SGLT1 upregulation [44,47].

Interestingly, we observed that fructose consumption induced a marked increase in *TAS1R2* and *TAS1R3* gene expression levels, associated with an increase in *SGLT1* and *GLUT2* expression levels. The close association between increased *TAS1R2/TAS1R3* and *SGLT1/GLUT2* expression levels suggests that fructose induces a TAS1R-dependent increase in SGLT1- and GLUT2-mediated sugar absorption. Moreover, the observation that both CP and SK were able to efficiently reduce *TAS1R2*, *TAS1R3*, *SGLT1* and *GLUT2* mRNA levels not only reinforces this conclusion but also allows us to conclude that the inhibitory effect of these two by-products upon these transporters is possibly mediated by *TAS1R2* and *TAS1R3* repression. Taken together, the above results suggest that the two coffee by-products, by decreasing the capacity of luminal sugar detection, limit the intestinal absorption of fructose, resulting in a decrease in postprandial fructose load. However, further functional verification in future studies is warranted, as no direct causality could be established.

Glucose- and fructose-derived metabolites activate the transcription factor ChREBP (Carbohydrate Response Element-Binding Protein), which regulates glucose and fructose metabolism through gene expression, thus underlying the effects of carbohydrates on glucose and lipid metabolism. In the liver, ChREBP overexpression causes fatty liver and lowers plasma glucose levels, and ChREBP deletion prevents obesity and fatty liver [49]. At the intestinal level, ChREBP regulates glucose and fructose absorption and catabolism by inducing sugar intestinal transporters (GLUT2 and GLUT5) and several genes involved in glycolysis, fructolysis and lipogenesis [50,51]. Interestingly, ChREBP^−/−^ mice are intolerant to a high sucrose and fructose diet, resulting in death [52,53].

SREBP-1c (Sterol Regulatory Element-Binding Protein-1c) is a transcription factor involved in transcriptional activation of genes involved in fatty acid and triglyceride synthesis, in response to hormonal and nutritional stimuli. It is present primarily in the liver, muscle and adipose tissue, but is also present in the small intestine [54]. Similar to other metabolic tissues, intestinal SREBP-1c links nutrient availability (postprandial state) to lipid metabolic gene expression [55]. In obese animal models, increased de novo lipogenesis and glycolysis pathways are observed in an intestinal epithelial cell line, as a consequence of SREBP-1c activation, and increased levels of hepatic SREBP-1c are also observed in obese patients [56,57]. In contrast, polyunsaturated fatty acids and polyphenolic-rich extracts inhibit SREBP-1c, thus reducing TAG overproduction and potential metabolic complications associated with obesity [58,59]. Moreover, a role of SREBP-1c in sucrose-induced hepatic steatosis was recently described in [60], which found that the gut microbiota and SREBP-1c are essential for sucrose-induced de novo lipogenesis and hepatic steatosis.

From the findings mentioned above, we decided to evaluate the effect of CP and SK on jejunal *ChREBP* and *SREBP-1c* gene expression. In our animals, high-fructose feeding resulted in a marked increase in jejunal *ChREBP-1* and *SREBP-1c* mRNA levels, and while SK was able to completely mitigate this effect, CP was ineffective. Therefore, the favorable metabolic effects of SK in fructose-fed animals may involve suppression of fructose-induced activation of these two transcription factors. We had previously verified that CP and SK did not interfere with hepatic *SREBP-1c* mRNA levels [16,17]. Therefore, the repressive effect of SK on *SREBP-1c* gene expression appears to be intestine-specific. Overall, it appears that, under high-fructose feeding, CP interferes with the intestinal sensing and absorption of sugars, while SK has a dual effect, limiting not only intestinal sensing and absorption of sugars but also fructose-induced SREBP-1c and ChREBP activation. However, as stated above, confirmation of these transcriptional findings at the functional level is needed.

Finally, we characterized the gut microbiota of these animals because it is known that the gut microbiota has the ability to influence host energy balance, metabolism and intestinal permeability [61,62]. No significant differences in the abundance of most represented phyla and genera and no changes in the *Firmicutes*/*Bacteroidetes ratio* were found between the groups of animals. The absence of changes in the taxa abundance of FRU rats was particularly unexpected. Indeed, in animal experiments, MetS induced by high-fructose feeding has been associated with an increase in the *Firmicutes* to *Bacteroidetes* ratio and with changes in the relative abundance of several bacterial species in the ileum, colon and feces (e.g., an increase in the relative abundance of *Proteobacteria* and *Firmicutes)*. However, these animal studies have also shown that the alterations in gut bacterial pattern inflicted by high-fructose chronic consumption are inconsistent, not only between species but also within species [63]. This may be related to the fact that the intestinal microbiota composition may vary considerably between animal facilities, being affected not only by the composition of the diet but also by prior treatment of the diet (e.g., irradiation), the bedding used in cages, and other housing conditions, as well as analytical methods used (e.g., analysis of DNA or RNA, extraction methods) [64]. It may also be dependent on the study design (e.g., the amount of fructose added and duration of treatment), and our own results support this assumption, as we observed changes in *E. coli* and *Lactobacillus* in rats of the same strain after drinking water with a lower amount of fructose (10%) for a much longer period (18 weeks) [20]. This lack of consistency of fructose feeding on the intestinal microbiota is also apparent in humans, where alterations of intestinal microbiota related to the consumption of a fructose-rich diet are even less clear and at times contradictory, which might be related to the different study locations, ethnicity and age of subjects studied as well as differences in study design [63]. Of note, the lack of significant effects of fructose on the microbiota is consistent with the observed phenotype of the animals: contrary to high-fat animal models, which develop obesity and are characterized by an increase in *Firmicutes* to *Bacteroidetes* ratio [65], the high-fructose animal models do not always develop obesity [5] and fructose-fed animals do not always display an increase in *Firmicutes* abundance, e.g., [66]. Again, the effects of fructose appear to be dependent on the vehicle of delivery (chow vs. water), dosage, duration, and the specific section of the intestine evaluated.

However, a significant difference was found in the abundance of the *Blautia* genus (one of the 25 most expressed genera), which increased in FRU animals, and a reduction in the FRU-induced increase was observed in CP + FRU animals. A similar stimulatory effect of fructose feeding on the relative abundance of *Blautia* was previously found in piglets [67]. The correlation between *Blautia* and metabolic health is ambiguous. On the one hand, this genus includes short-chain fatty acid-producing bacteria, but on the other hand, an increase in its abundance has been associated with adverse metabolic profiles, namely hypertriglyceridemia, insulin resistance and visceral fat deposition [68,69]. In this context, the phenotype of the FRU animals (high blood pressure, insulin resistance, hypertriglyceridemia) suggests an association between the increase in *Blautia* abundance in FRU animals and their metabolic derangement. Dietary fructose feeds hepatic lipogenesis via microbiota-derived acetate [70] and gut microbiota-derived acetate can regulate blood pressure [71]. Because members of the genus *Blautia* are important acetate-producing bacteria in the human gut [72], we can speculate that fructose-induced stimulation of intestinal *Blautia* spp. contributes to increased hepatic lipogenesis and hypertension in FRU animals. Of note, the fructose-induced increase in *Blautia* abundance was reversed by CP, but not by SK, which correlates with the more pronounced inhibitory effects of CP on fructose-induced metabolic alterations, in relation to SK, that we observed [16,17].

Moreover, although statistically not significant, the abundance of *Ligilactobacillus* tended to be smaller in FRU animals. The *Ligilactobacillus* genus, which results from a recent taxonomic reclassification of the *Lactobacillus* genus and constitutes one of the major groups of commensal bacteria of healthy rodents [73], was the most abundant in CONT animals. *Ligilactobacillus* spp. contribute to host homeostasis by fermenting carbohydrates into lactic acid that inhibits harmful microbes, thus modulating microbial community structure, enhancing epithelial barrier integrity, and supporting immune function [74]. Interestingly, both coffee by-products tended to increase the abundance of *Ligilactobacillus*, suggesting a potential preservation of this beneficial genus and reversal of this specific microbial trend.

In summary, no major significant differences in the relative abundance of the most common phyla and genera of bacteria were observed between the groups. However, CP was able to reverse the increase in *Blautia* spp. abundance induced by fructose feeding.

In relation to microbial diversity, a singular pattern was found. The FRU group of animals exhibited the highest values of richness (*Chao1*) and diversity (*Shannon*) among all groups. The increase in microbial diversity may reflect ecological disruption through overgrowth, as it is possible that elevated luminal fructose provides additional metabolic niches, promoting the proliferation of opportunistic sugar-fermenting bacteria. Therefore, while this transiently increases species richness, it compromises functional homeostasis and intestinal barrier integrity [75,76]. Importantly, both CP and SK mitigated fructose-induced increase in microbial richness (*Chao1*). In addition, SK prevented fructose-induced increase in microbial diversity (*Shannon*), whereas CP showed a trend toward reducing this parameter.

However, analysis of beta diversity (PCoA) showed distinct effects of CP and SK: although CP induced a significant modulation of the bacterial community structure, the same was not observed with SK. Thus, this observation reinforces the conclusion that CP, but not SK, is able to modulate the gut microbial community. Of note, because sequencing of V3 and V4 regions of the 16S rRNA gene was performed in the present study, it was possible to observe dynamic changes in beta diversity and the identification of specific taxa at the genus level, thereby providing an analytical resolution that has not been addressed in a previous investigation involving this coffee by-product [77].

There is a link between gut microbiota and host SREBP-1c and ChREBP, mediated by microbiota-derived metabolites, inflammatory signaling, and alterations in bile acid metabolism [78,79]. Although SK did not induce significant changes in overall microbial beta diversity, modulation of these transcription factors may occur through microbiota-independent mechanisms (e.g., by a direct effect of SK components such as caffeine) or through alterations in microbial metabolic activity that are not captured by taxonomic beta diversity analyses.

An important point to discuss is the fact that, although CP and SK possessed similar effects at the intestinal level (namely, abrogation of fructose-induced increase in jejunal *SGLT1*, *GLUT2*, *TAS1R2* and *TAS1R23* mRNA levels), these two by-products also exhibited some differential effects: CP was able to modulate fructose-induced alterations in the gut microbiota, whereas SK mitigated fructose-induced changes in the jejunal expression of transcription factors involved in carbohydrate and lipid metabolism. These differences are consistent with their distinct effects on fructose-induced MS-related parameters [16,17], and are probably related to differences in their composition. In particular, variations in dietary fiber content (SK being richer in dietary fiber) and type (soluble fiber consisting mainly of pectins in SK but of xyloglucans, mannans, and pectins in CP), free sugars (CP being richer in free sugars—glucose and fructose), proteins (more abundant in SK), and caffeine and chlorogenic acids (more abundant in SK) (Supplementary Table S1) [80,81] may be involved, as these dietary components are known to influence the host gut microbiota and carbohydrate and lipid metabolism, e.g., [82,83]. Additionally, CP, being a fresh (unroasted) by-product, contains an open carbohydrate matrix with free sugars [84], which serves as a highly accessible carbon source in the lumen, promoting glycan-degrading commensals like *Blautia* via a prebiotic ‘bulk effect’ [85]. Conversely, roasting binds SK fibers into complex, stable ‘melanofibers’ resistant to rapid fermentation [86]. Consequently, the therapeutic value of SK probably relies on its concentrated bioactives (chlorogenic acids, caffeine, and melanoidins) escaping the matrix [86]. This contrast between CP luminal prebiotic action and SK direct cellular signaling highlights how upgrading distinct coffee by-products can target specific pathological axes of MS.

Finally, we must acknowledge some limitations of this study. First, the core active components responsible for the effects of CP and SK were not identified, and dietary fiber may act as a confounding factor. Second, some interanimal variability in the response to the coffee by-products was observed. Third, the single-dose gavage protocol precludes the evaluation of a dose–response relationship. Finally, a relatively small sample size (*n* = 6 animals per group) was used, which may have reduced statistical power to detect modest differences and increased the possibility of Type II error.

## 5. Conclusions

This work characterized the consequences of excessive fructose consumption at the intestinal level and explored the potential of two coffee by-products in ameliorating these consequences by using a validated experimental rat model of MetS [14,15] and establishing integrated morphological, biochemical and microbiological signatures. We verified that fructose consumption did not cause significant morphological alterations in duodenal and jejunal mucosa. However, it caused a very marked increase in the gene expression levels of sugar transporters (*SGLT1*, *GLUT2*), sweet taste receptors (*TAS1R2*, *TAS1R3*) and transcription factors (*SREBP-1c* and *ChREBP*) in the jejunum of these animals. Moreover, it induced a pattern of dysbiosis distinct from that observed in fat-induced obesity. Namely, no changes in the *Firmicutes*/*Bacteroidetes* ratio were observed; rather, an excessive growth of certain taxa, resulting in an increase in alpha diversity, associated with an increase in the abundance of *Blautia* spp. and a tendency for a reduction in the abundance of *Ligilactobacillus*, was observed. Moreover, this study showed that CP and SK were able to abrogate fructose-induced increases in jejunal *SGLT1*, *GLUT2*, *TAS1R2* and *TAS1R23* mRNA levels. Additionally, CP modulated fructose-induced alterations in gut microbiota, whereas SK mitigated fructose-induced changes in the jejunal expression of transcription factors involved in carbohydrate and lipid metabolism.

Overall, our findings suggest that CP and SK may represent promising functional ingredients for mitigating fructose-induced intestinal alterations and support their use in functional foods targeting MS. Valorization of these by-products may also contribute to a circular economy. However, clinical studies are needed to validate their health benefits in humans, and future research should focus on optimizing and standardizing CP and SK extracts to enhance reproducibility and industrial application.

## Data Availability

The original contributions presented in this study are included in the article and Supplementary Materials. Further inquiries can be directed to the corresponding author.

## Supplementary Materials

The following supporting information can be downloaded at: https://www.mdpi.com/article/10.3390/biom16071069/s1, Table S1: Chemical composition of the standard feed, dried coffee pulp (CP) and dried coffee silverskin (SK); Table S2: Primer sequences and annealing temperature (AT) used in gene expression analysis by qRT-PCR.

## Abbreviations

The following abbreviations are used in this manuscript:

CONT	Control
FRU	Fructose
CP	Coffee pulp
SK	Coffee silverskin
