# Supplementary material for: Coffee Pulp and Silverskin Mitigate Fructose-Induced Intestinal Alterations in Rats
Source: Biomolecules. 2026 Jul 22;16(7):1069. doi: 10.3390/biom16071069 (PMC13406555; doi:10.3390/biom16071069)
Supplement: Supplementary file 1 [file biomolecules-16-01069-s001.zip › biomolecules-4402855-supplementary.pdf]

Supplementary Material

Supplementary Table S1. Chemical composition of the standard feed, dried coffee pulp (CP) and dried coffee silverskin (SK).

Analyzed parameters	Feed	CP[1]	SK[2]
Moisture (g/100 g)	8.95 ± 0.40	12.07 ± 0.57	8.06 ± 0.20
Ash (g/100 g)	6.53 ± 0.09	6.34 ± 0.36	9.65 ± 0.01
Crude Protein (g/100 g)	21.03 ± 0.38	7.00 ± 0.19	16.29 ± 0.20
Total Fat (g/100 g)	2.76 ± 0.01	0.89 ± 0.08	1.97 ± 0.01
Total Carbohydrates (g/100 g)	69.68 ± 0.45	85.77 ± 0.23	72.09 ± 0.20
Total Dietary Fiber (g/100 g)	21.30 ± 0.46	38.51 ± 0.13	70.21 ± 0.24
Insoluble Dietary Fiber (g/100 g)	17.05 ± 1.01	31.69 ± 0.46	54.86 ± 0.70
Soluble Dietary Fiber (g/100 g)	4.25 ± 0.54	6.82 ± 0.59	15.35 ± 0.47
Remaining Carbohydrates (g/100 g)	48.38 ± 0.84	47.26 ± 0.16	1.88 ± 0.42
Energy (kcal/100 g)	348.89 ± 1.20	304.24 ± 1.14	233.16 ± 0.37
Energy (kJ/100 g)	1468.68 ± 5.35	1272.66 ± 4.86	953.36 ± 1.72
Fatty acids (relative %)			
C14:0	0.87 ± 0.04	1.22 ± 0.08	1.99 ± 0.17
C16:0	15.31 ± 0.12	36.95 ± 1.56	24.84 ± 0.83
C16:1	0.95 ± 0.03	n.d.	n.d.
C18:0	2.89 ± 0.19	7.10 ± 0.62	7.75 ± 0.44
C18:1n9c	25.36 ± 0.04	20.83 ± 4.97	12.89 ± 0.20
C18:2n6c	47.93 ± 0.25	20.66 ± 1.72	20.50 ± 0.16
C18:3n3	4.10 ± 0.05	13.25 ± 1.15	2.09 ± 0.14
C20:0	n.d.	n.d.	14.98 ± 0.67
C20:1n9	0.77 ± 0.06	n.d.	n.d.
C20:5n3	0.80 ± 0.01	n.d.	n.d.
C22:0	n.d.	n.d.	14.96 ± 0.99
C22:6n3	1.01 ± 0.08	n.d.	n.d.
Caffeine (g/100 g)	n.d.	0.50 ± 0.01	0.89 ± 0.01
Chlorogenic acids (mg/100 g)			
3-caffeoylquinic acid	n.d.	2.17 ± 0.23	18.56 ± 1.71
5-caffeoylquinic acid	n.d.	133.12 ± 2.26	164.16 ± 3.11
4-caffeoylquinic acid	n.d.	6.24 ± 0.33	38.16 ± 1.28

Results are expressed per 100 g of dry weight. Abbreviations: n.d., not detected

[1] The composition of CP was originally described by [17].

[2] The composition of SK was originally described by [16].

Supplementary Table S2. Primers sequences and annealing temperature (AT) used in gene expression analysis by qRT-PCR.

Gene name	Primer Sequence (5'-3')	AT
GAPDH	**F -** GGCATCGTGGAAGGGCTCATGAC<br>**R -** ATGCCAGTGAGCTTCCCGTTCAGC	70 °C
SGLT1	**F -** GGGTGGCTTTTTGTCCCTAT<br>**R -** GGTAGATCTGGATCCGCTTG	59 °C
GLUT2	**F -** TTCTGTGCCGTCTTCATGTC<br>**R -** TGGCCCAATCTCAAAGAAAC	59 °C
GLUT5	**F -** GAAAAGCAACGACGTCCAAT<br>**R -** CCCCAAAGCTCTACCACAAA	59 °C
TAS1R2	**F -** TGGACTTGCTGCTGTCTGTG<br>**R -** GGAGATGGAGGAGGTGAAGG	60 °C
TAS1R3	**F -** GTGGGTCACTGTTCTGCTTTGG<br>**R -** GGAGGTGAGCCATTGGTTGTT	60 °C
SREBP-1c	**F -** GTGGGTCTCCTCCGAAGCCG<br>**R -** AGCATGTCTTCGATGTCGGTCAAG	60 °C
ChREBP	**F -** GTGGCCATGGCGCGCGCGCTGGCGGATC<br>**R -** AGGATTATAATGGTCTCCCCAGGGTGCC	60 °C

Abbreviations: GAPDH: Glyceraldehyde-3-Phosphate Dehydrogenase, SGLT1: Sodium-Glucose Linked Transporter 1, GLUT2: Facilitative Glucose Transporter Type 2; GLUT5: Facilitative Glucose Transporter Type 5, TAS1R2; Taste Receptor Type 1 Member 2, TAS1R3; Taste Receptor Type 1 Member 3, SREBP-1c: Sterol Regulatory Element-Binding Protein 1c, ChREBP: Carbohydrate Response Element-Binding Protein, F: forward, R: reverse.
